# Nanomaterials for the Prevention, Detection, and Treatment of Pharyngeal Human Papillomavirus Infection: A Translational Roadmap

**DOI:** 10.3390/ma19153187

**Published:** 2026-07-26

**Authors:** Lorena Adriana Paun, Mihai Dumitru, Diana Gabriela Iacob, Oana Maria Patrascu, Daniela Vrinceanu, Rares Oanca, Alexandru-Darius Dragomir-Serboiu, Andreea Marinescu, Monica-Mihaela Cirstoiu

**Affiliations:** 1 ENT Department, Carol Davila University of Medicine and Pharmacy, 020021 Bucharest, Romania; paunlorena98@yahoo.com (L.A.P.); vrinceanudana@yahoo.com (D.V.); oanca.rares@yahoo.com (R.O.); alexandru-darius.dragomir2024@stud.umfcd.ro (A.-D.D.-S.); 2 Epidemiology and Infectious Diseases Department, Carol Davila University of Medicine and Pharmacy, 020021 Bucharest, Romania; diana.iacob@umfcd.ro; 3 Pathology Department, Carol Davila University of Medicine and Pharmacy, 020021 Bucharest, Romania; oanamaria.patrascu@gmail.com; 4 Radiology and Imaging Department, Carol Davila University of Medicine and Pharmacy, 020021 Bucharest, Romania; 5 Gynecology Department, Carol Davila University of Medicine and Pharmacy, 020021 Bucharest, Romania; monica.cirstoiu@umfcd.ro

**Keywords:** biomaterials, nanomaterials, nanoparticle characterization, structure–property–function, mucosal delivery, HPV, oropharyngeal, lipid nanoparticle platforms, polymeric nanoparticle platforms, theranostics

## Abstract

Pharyngeal infection with high-risk human papillomavirus (HPV), particularly HPV16, is biologically distinct from cervical infection because it occurs within the specialized lymphoepithelial environment of Waldeyer’s ring. This review evaluates nanoparticle materials for the prevention, detection, and treatment of pharyngeal HPV, with an emphasis on structure–property–function relationships, mucosal performance, and translational feasibility. Lipid nanoparticle platforms, polymeric nanoparticle platforms, inorganic systems, and hybrid platforms are compared with respect to composition, particle size distribution, surface charge, colloidal stability, biodegradability, payload compatibility, release behavior, and manufacturing complexity. Evidence suggests that lipid and polymeric systems are the most credible near-future candidates for mucosal vaccination and localized nucleic acid delivery because they offer the best balance between controllable fabrication, analytical tractability, and biologically plausible performance in mucus-exposed tissue. By contrast, the development of inorganic theranostics and CRISPR-enabled platforms remains at an earlier stage because repeated mucosal dosing, retention in lymphoid tissue, and combined product regulation impose substantial burdens. A translational roadmap is proposed in which material selection is guided by clinically relevant quality attributes, standardized saliva- and mucus-relevant assays, human tonsil organoid testing, and early attention to manufacturability, safety, and regulatory strategy. The field is promising, but direct pharyngeal HPV data remain limited; accordingly, there is an urgent need for comparative studies that connect nanoparticle architecture to measurable outcomes such as tonsillar deposition, epithelial uptake, immune activation, and local tolerability.

## 1. Introduction

HPV-associated disease of the oropharynx has become an important clinical and public health concern, especially as the incidence of HPV-positive oropharyngeal squamous cell carcinoma (OPSCC) has increased in many populations [1]. Although prophylactic vaccination has altered the long-term approach to prevention, persistent pharyngeal infection, early mucosal lesions, and localized therapeutic intervention remain insufficiently addressed [2]. The oropharynx is not simply another mucosal surface; tonsillar crypts, a discontinuous epithelium, lymphoid-rich tissue, saliva exposure, and intense immune surveillance create a microenvironment in which transport, retention, antigen sampling, and inflammatory responses differ from those observed in the cervix or lower respiratory tract [3].

These site-specific features make pharyngeal HPV a compelling target for nanomaterials, but they also present a challenging design problem. A useful formulation must function in mucus-exposed tissue, tolerate rapid fluid turnover, interact appropriately with epithelial and immune cells, and remain manufacturable at a clinically realistic scale [4]. For this reason, the central question is not whether nanoparticle platforms can deliver cargo in principle but which material classes offer robust structure–property–function relationships that can be measured, optimized, and reproduced [5,6]. Parameters such as particle size distribution, polydispersity, ionization behavior, surface chemistry, hydration layer, degradability, and release kinetics are therefore not secondary formulation details; they are determinants of deposition in tonsillar crypts, epithelial uptake, local immune programming, and translational feasibility [7,8].

Current strategies for controlling HPV-associated disease already include several complementary approaches for prevention, detection, and treatment, although their clinical maturity differs considerably across anatomical sites. The primary preventative approach is prophylactic HPV vaccination, which is most effective before viral exposure and prevents new infections rather than clearing established infections or treating HPV-related lesions. Vaccination programs are supported by public health education, risk-reduction counselling, condom or barrier protection, smoking cessation, and efforts to improve vaccine uptake across both sexes. At the population level, the most developed prevention framework remains cervical cancer elimination, in which high vaccine coverage is combined with high-performance screening and treatment of detected precancerous disease. However, comparable prevention pathways are not yet available for pharyngeal HPV because infection is often asymptomatic, the relevant mucosal reservoir is difficult to sample reproducibly, and validated premalignant screening targets for the oropharynx remain poorly defined [9].

Detection strategies presently range from established cervical screening tests to emerging oropharyngeal biomarkers. In cervical disease, HPV DNA testing, partial or extended genotyping, cytology-based triage, dual-stain cytology, colposcopy, and histopathology form structured algorithms that can identify clinically relevant precancer and guide treatment. For oropharyngeal disease, p16 immunohistochemistry is widely used as a practical surrogate marker of HPV-driven carcinogenesis in OPSCC, but confirmatory HPV DNA, RNA in situ hybridization, polymerase-chain-reaction-based assays, or E6/E7 mRNA testing may be needed when diagnostic certainty is important, particularly for staging, prognosis, and treatment de-escalation decisions. Non-invasive or minimally invasive approaches such as oral rinse, saliva, or swab-based HPV DNA detection; HPV16 E6 serology; circulating tumor HPV DNA; next-generation sequencing; digital PCR; and artificial-intelligence-assisted imaging or pathology analysis are under active investigation. These approaches may improve risk stratification and surveillance, but none currently provide a validated, population-level screening program for asymptomatic pharyngeal HPV infection [10].

Treatment strategies are similarly site- and stage-dependent. Cervical precancer can be treated through ablative or excisional procedures within organized screening programs, while benign oral HPV-related lesions may be managed by observation, conservative excision, laser therapy, cryotherapy, or other local destructive techniques depending on lesion type and symptoms. For HPV-positive OPSCC, care comprises combinations of transoral surgery, radiotherapy, chemotherapy, and selected immunotherapy approaches in recurrent or metastatic disease. Because HPV-positive OPSCC often has a favorable prognosis, treatment de-intensification is being explored to reduce long-term morbidity, but de-escalation must be carefully validated because inadequate treatment may compromise disease control. Therapeutic vaccines, immune-checkpoint combinations, adoptive cellular therapies, RNA interference, antisense oligonucleotides, genome editing, photodynamic therapy, and image-guided focal interventions are promising research directions, yet most remain investigational or restricted to defined oncologic contexts rather than the routine management of persistent pharyngeal HPV infection [11].

Together, these existing strategies demonstrate that HPV control is already multifaceted, but they also expose important translational gaps at the pharyngeal mucosal interface: vaccines prevent future infection but do not eliminate established mucosal HPV; while cervical screening pathways are mature, validated oropharyngeal screening remains absent; and standard OPSCC treatments address established malignancy rather than early, localized viral persistence. These limitations justify interest in technologies that can improve local mucosal retention, protect labile nucleic acid or protein payloads, enhance antigen presentation, support earlier lesion mapping, or combine diagnostic and therapeutic functions. Nanomaterial platforms should therefore be considered complementary to vaccination, screening, and standard oncologic care rather than substitutes for them, and their translational value will depend on whether they offer measurable advantages over simpler approaches in prevention, detection, or treatment [12].

This review provides a concise, material-focused assessment of nanoparticle platforms for the prevention, detection, and treatment of pharyngeal HPV. Rather than cataloging every reported formulation, lipid, polymeric, inorganic, and hybrid systems are compared in terms of fabrication, physicochemical characterization, mucosal delivery performance, and translational maturity. Particular emphasis is placed on where direct evidence exists for pharyngeal use, where conclusions rely on analogy from adjacent mucosal fields, and which experimental and manufacturing gaps must be resolved before clinical development is credible.

## 2. Materials and Methods

This article is a narrative review designed for a materials science readership. Its purpose is to identify nanoparticle design principles that are most relevant to pharyngeal HPV prevention, detection, and therapy rather than to provide a formal systematic review or meta-analysis. The analysis prioritizes composition, fabrication route, particle architecture, colloidal behavior, payload compatibility, release characteristics, mucoadhesion or mucus penetration, and manufacturability [13].

Studies were identified through targeted searches of PubMed, Web of Science, and major publisher databases, with emphasis on publications from 2020 to early 2026 and selective inclusion of older foundational studies where necessary. The search terms combined disease, anatomical site, payload, and materials-engineering concepts, including “HPV”, “oropharyngeal”, “tonsil”, “mucosal vaccine”, “nanoparticle”, “lipid nanoparticle”, “polymeric nanoparticle”, “mucoadhesion”, “siRNA”, “antisense oligonucleotide”, “CRISPR”, “theranostic”, “scale-up”, and “quality by design”. Studies were favored when they reported formulation details, characterization methods, biological performance in mucosal or head and neck models, or translational issues such as stability, sterility, storage, toxicity, or manufacturing feasibility.

Because there have been few direct nanoparticle studies in pharyngeal HPV, relevant evidence was also drawn from mucosal vaccinology, head and neck oncology, respiratory delivery, and nanomedicine for other epithelial viral targets. Such extrapolation is useful but imperfect [14].

Throughout this review article, claims are therefore weighted according to how closely the available model reflects the intended pharyngeal application. Greater emphasis is placed on studies that help answer four translational questions: Which material properties govern performance at the pharyngeal mucosal interface? Which payload-platform pairings are most mature? Which preclinical models are most informative? Finally, which manufacturing or regulatory barriers are likely to determine success or failure?

## 3. Platform Landscape and Comparative Analysis

We compared the main nanoparticle platforms being considered for pharyngeal HPV prevention, detection, and treatment, with an emphasis on how material properties shape translational potential [15,16]. Lipid and polymeric nanoparticle platforms emerge as the most credible options in the near future because they offer better control over size, surface behavior, payload compatibility, and manufacturing reproducibility. Inorganic and hybrid systems provide valuable imaging, triggered-release, and theranostic functions, but they remain more complex and less mature for routine pharyngeal use [17,18]. Across all platforms, the key message is that clinically relevant progress depends on linking physicochemical attributes to mucosal performance, local tolerability, and scalable production rather than on increasing technological complexity alone.

### 3.1. Comparative Overview of Nanoparticle Platforms

Among the classes currently available, lipid and polymeric nanoparticle platforms present the strongest translational logic because their critical quality attributes can be linked, at least in principle, to mucosal performance and manufacturing control [19]. Lipid nanoparticle platforms are especially attractive for mRNA and siRNA delivery owing to established ionizable-lipid design frameworks and scalable mixing processes, but their performance in saliva-rich pharyngeal tissue and their tolerability after repeated local dosing remain insufficiently defined [20]. Polymeric systems offer greater flexibility for mucoadhesion and sustained exposure, which is advantageous for local application to tonsillar tissue, yet the same adhesive properties can reduce reproducibility, complicate release testing, and increase the risk of irritation [21]. Inorganic and hybrid platforms offer valuable imaging, triggered-release, and multifunctional capabilities, but the analytical burden is higher and the justification for routine pharyngeal use is currently weaker [22]. In short, the most plausible near-future products are not the most technologically elaborate ones but the systems for which material properties can be controlled, measured, and matched to a specific clinical use case, as reported in Table 1.

### 3.2. Mucosal Vaccination Strategies for the Pharynx

**Figure 1:**
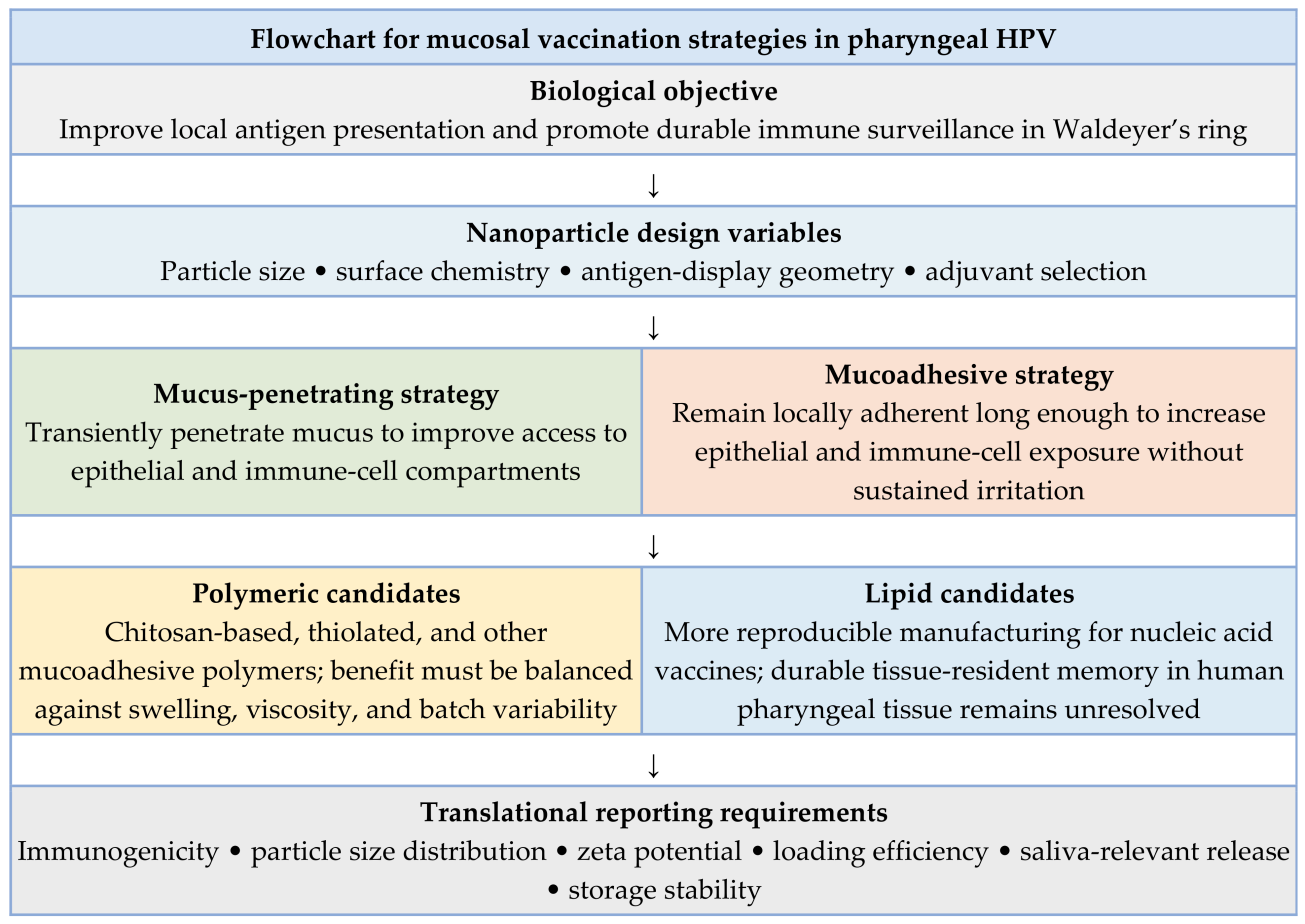
Flowchart summarizing mucosal vaccination strategies for pharyngeal HPV. Nanoparticle design begins with the goal of improving local antigen presentation and durable immune surveillance in Waldeyer’s ring and then proceeds according to formulation variables, including particle size, surface chemistry, antigen-display geometry, and adjuvant selection. Mucus-penetrating systems aim to enhance access to epithelial and immune-cell compartments, whereas mucoadhesive systems seek prolonged local exposure without sustained irritation. Polymeric candidates such as chitosan-based or thiolated materials and lipid candidates for nucleic acid vaccination should be advanced only with clear reporting of immunogenicity, particle size distribution, zeta potential, loading efficiency, saliva-relevant release, and storage stability.

Mucosal vaccination is one of the most attractive applications of nanoparticle materials in pharyngeal HPV because the biological goal—to improve local antigen presentation and promote durable immune surveillance in Waldeyer’s ring—is well-defined [23]. Particle size, surface chemistry, and antigen-display geometry likely influence mucus transport, dendritic-cell access, and cross-presentation, whereas adjuvant selection shapes the balance between protective immunity and excessive local inflammation [24]. The strongest rationale lies in formulations that can either transiently penetrate mucus or remain locally adherent long enough to increase epithelial and immune-cell exposure without causing sustained irritation [25]. Chitosan-based, thiolated, and other mucoadhesive polymers are appealing in this regard, but their benefit must be weighed against variability in swelling, viscosity, and batch-to-batch performance [26]. Lipid systems may offer more reproducible manufacturing for nucleic acid vaccines, yet the extent to which they induce truly durable tissue-resident memory responses in human pharyngeal tissue remains unresolved [27,28]. For translation, vaccine studies should report not only immunogenicity but also particle size distribution, zeta potential, loading efficiency, release under saliva-relevant conditions, and stability after storage, as summarized in Figure 1 [29,30].

### 3.3. Nucleic Acid Therapeutics and Genome Editing

Nucleic acid therapeutics are particularly relevant to HPV because viral oncogene expression offers a direct molecular target [31]. Small interfering RNA and antisense oligonucleotides aimed at E6/E7 transcripts are conceptually attractive for localized pharyngeal delivery because they can suppress oncogenic signaling while limiting systemic exposure if retained near the mucosal target [32]. In these circumstances, lipid nanoparticle platforms and selected polymeric carriers are the most plausible options because they protect cargo from nuclease degradation and can be formulated for transient epithelial uptake [33]. However, translation depends on more than knockdown efficiency. Saliva stability, mucus interaction, release rate, local tolerability, and repeat-dose feasibility are all likely to determine whether a candidate is clinically usable [34,35].

**Figure 2:**
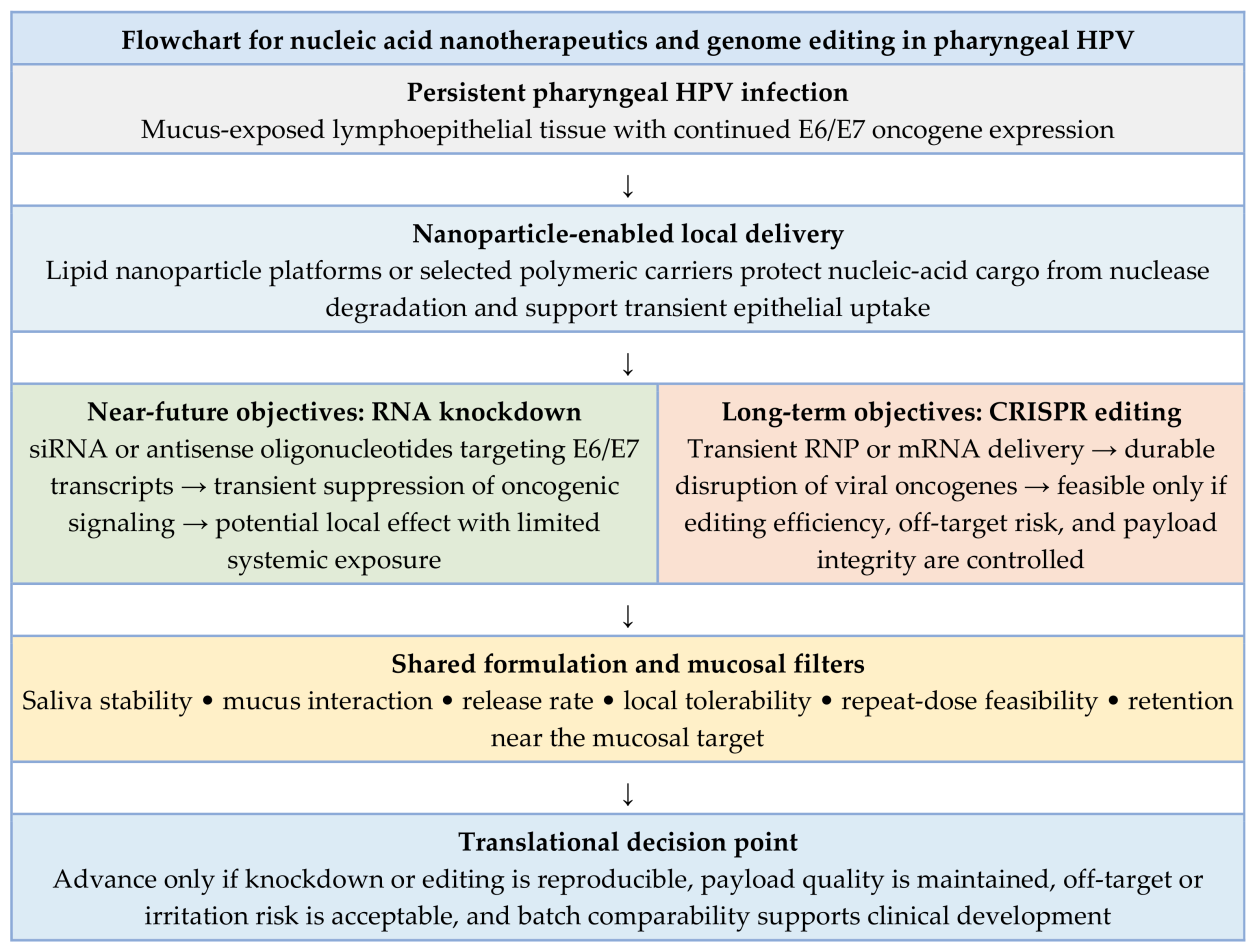
Flowchart summarizing nanoparticle-enabled nucleic acid strategies for persistent pharyngeal HPV infection. Local delivery systems are designed to protect siRNA, antisense oligonucleotides, CRISPR ribonucleoproteins, and mRNA from nuclease degradation while supporting transient epithelial uptake near E6/E7-expressing mucosal targets. RNA knockdown is the near-future objective, whereas CRISPR-based disruption of viral oncogenes remains a long-term goal requiring stringent control of editing efficiency, off-target risk, payload integrity, local tolerability, repeat-dose feasibility, and batch comparability.

CRISPR-based strategies move one step further by seeking durable disruption of viral oncogenes, but they also move further away from near-future clinical reality. For pharyngeal use, transient nanoparticle delivery of ribonucleoproteins or mRNA is preferable to persistent expression systems because it reduces the risk of prolonged nuclease activity [36]. Efficient editing in lymphoepithelial tissue, rigorous off-target analysis, payload integrity testing, and robust batch comparability are all necessary for defensible human translation [37]. At present, CRISPR nanomedicine should be viewed as a longer-term option whose feasibility of which depends on advances in targeted local delivery and improved safety analytics rather than as an immediate solution to persistent pharyngeal HPV (Figure 2) [38].

### 3.4. Mucoadhesive Local Delivery and Dosing Paradigms

Local delivery to the oropharynx is attractive because it can increase the drug concentration at the intended site while reducing systemic exposure, but formulation design is constrained by anatomy and patient use [39]. Sprayable suspensions, mucoadhesive gels, and dissolvable films each have trade-offs in terms of residence time, coverage, comfort, and compatibility with nanoparticle stability [40]. Strong mucoadhesion may improve exposure within the tonsillar crypts, yet excessive viscosity can impair dose reproducibility and user acceptability. Conversely, rapidly diffusing low-viscosity systems may be easier to administer but might fail to provide sufficient contact time [41]. Translational studies should therefore evaluate not only drug release but also sprayability, rheology, storage stability, container compatibility, sterilization strategy, and clearance from the mucosal and lymphoid compartments under saliva-relevant conditions [42]. Such practical variables are often underreported, even though they are likely to determine whether promising preclinical formulations can become usable products [43].

**Figure 3:**
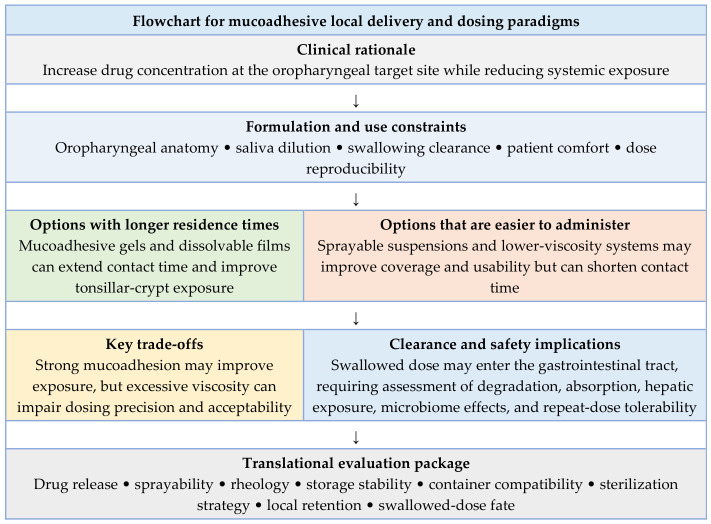
Flowchart summarizing mucoadhesive local delivery and dosing paradigms for oropharyngeal nanoparticle formulations. Local delivery is intended to increase drug concentration at the mucosal target while limiting systemic exposure, but actual performance depends on anatomy, saliva dilution, swallowing clearance, patient comfort, and dose reproducibility. Systems with longer residence times, such as mucoadhesive gels and dissolvable films, may improve tonsillar-crypt exposure, whereas sprayable suspensions and lower-viscosity systems may be easier to administer but can shorten contact time. Because part of the applied dose may be swallowed, translational development should evaluate drug release, sprayability, rheology, storage stability, container compatibility, sterilization strategy, local retention, and gastrointestinal fate after repeat dosing.

The expected differences in biodistribution between systemic administration and local mucoadhesive oropharyngeal delivery are summarized schematically in Figure 3. However, local application should not be interpreted as purely local exposure. Sprays, gels, films, and rinses applied to the mouth and throat are continuously diluted by saliva and are often cleared predominantly by swallowing. As a result, a meaningful fraction of the administered nanoparticle dose may enter the gastrointestinal tract, where particle degradation, payload release, absorption across the intestinal barrier, hepatic first-pass exposure, and microbiome interactions become relevant safety considerations. Figure 3 should therefore be read as a simplified comparison of intended deposition patterns rather than a complete pharmacokinetic map; translational studies should quantify both the local retention and gastrointestinal fate of swallowed doses, including gastrointestinal stability, biodegradation products, systemic absorption, and repeat-dose tolerability.

### 3.5. Theranostics for Lesion Mapping and Minimally Invasive Therapy

**Figure 4:**
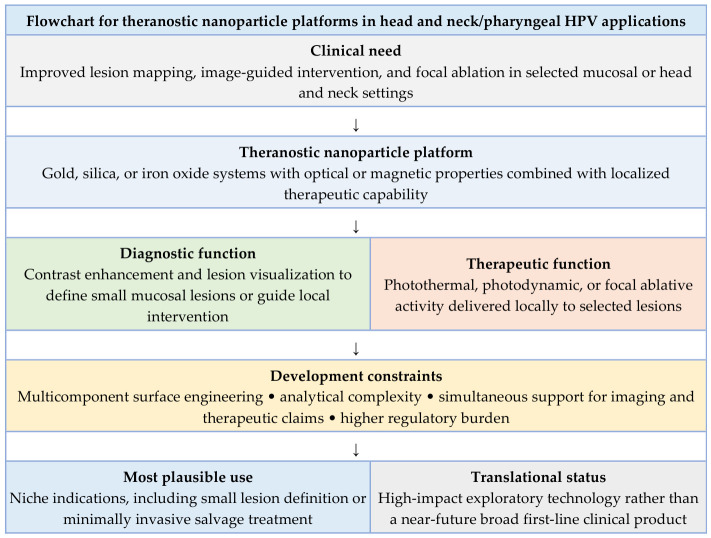
Flowchart summarizing theranostic nanoparticle concepts for lesion mapping and minimally invasive therapy in selected head and neck/pharyngeal HPV settings. Gold-, silica-, and iron oxide-based systems can combine diagnostic contrast with localized photothermal, photodynamic, or focal ablative functions, enabling image-guided intervention or improved definition of small mucosal lesions. Because these platforms require multicomponent surface engineering and must support both imaging and therapeutic claims, their near-future value is likely to remain focused on niche indications rather than broad first-line clinical use.

Theranostic nanoparticle platforms combine diagnostic contrast with localized therapy and are therefore attractive for lesion mapping, image-guided intervention, and focal ablation in selected head and neck settings [44]. Gold-, silica-, and iron oxide-based systems are especially relevant because their optical or magnetic properties can be paired with photothermal, photodynamic, or imaging functions [45]. These platforms are analytically complex, often rely on multicomponent surface engineering, and may face a higher regulatory burden because imaging and therapeutic claims must be supported simultaneously [46]. Their greatest value may lie in niche indications, such as defining small mucosal lesions or supporting minimally invasive salvage treatment, rather than broad first-line use [47]. At present, they are best regarded as high-impact exploratory technologies rather than near-future clinical products (Figure 4) [48].

## 4. Discussion

We discuss how nanoparticle concepts for pharyngeal HPV can move from promising laboratory formulations toward clinically realistic products, identifying the most credible short-term opportunities, especially mucosal vaccines and localized nucleic acid delivery and contrasting them with more complex platforms that remain longer-term prospects [49]. We also examine the major barriers that continue to limit translation, including inadequate biological models, incomplete safety assessment, and poor reproducibility across published systems. Finally, we propose a staged preclinical pathway in which formulation control, pharyngeal relevance, local tolerability, and measurable biological endpoints are established early to improve decision-making and reduce avoidable translational failure.

### 4.1. Translational Opportunities

The most credible near-future opportunities are formulations that solve a limited, clearly defined problem. Two examples stand out: mucosal vaccine systems intended to improve local immune priming in Waldeyer’s ring and localized siRNA or antisense formulations aimed at transient suppression of viral oncogenes. These strategies benefit from simpler product concepts, more established carrier classes, and clearer pharmacodynamic endpoints than multifunctional or gene-editing platforms [50]. A realistic medium-term goal is not immediate disease eradication but proof that a formulation can achieve safe local delivery, measurable retention or uptake, and a biological signal such as viral-load reduction, mucosal antibody production, or immune-cell activation [51]. By contrast, CRISPR-based products and integrated theranostics will likely remain longer-term prospects because their development requires more demanding targeting, analytics, and regulatory planning [52].

### 4.2. Principal Barriers and Mitigation Strategies

Three barriers dominate the field. First, delivery remains uncertain because human tonsillar anatomy is difficult to model and standard cell-line systems do not capture crypt architecture, mucus exposure, or immune complexity [53]. Second, safety is a central concern for any product intended for repeated mucosal or lymphoid exposure; local irritation, chronic immune activation, and tissue retention must be studied directly rather than inferred from systemic administration data [54]. Third, reproducibility remains a weak point across many published formulations. Studies often compare platforms without standardized characterization, making it difficult to know whether performance differences arise from chemistry, size distribution, surface properties, or uncontrolled process variation [55,56]. These problems can be mitigated by adopting predefined critical quality attributes, orthogonal analytical methods, human tonsil organoid screening, and early engagement with manufacturing and regulatory requirements [57]. In translational nanomedicine, simplicity is often an advantage: the platform with fewer uncontrolled variables is more likely to progress than the one with the longest feature list [58].

### 4.3. Recommended Preclinical Pipeline

A credible preclinical pipeline for nanoparticle interventions in pharyngeal HPV should begin with disciplined formulation screening rather than broad efficacy testing. In this first stage, investigators should define a clear target product profile, identify the critical quality attributes most likely to determine performance, and confirm payload integrity under manufacturing and administration-relevant conditions. Screening should move quickly beyond standard monolayer assays toward human tonsil organoids or similarly relevant ex vivo systems capable of capturing uptake, epithelial interaction, early immune signaling, and irritation potential in a lymphoepithelial context [59]. Once a candidate shows acceptable physicochemical control and biological plausibility, the next step is to establish biodistribution and retention in models that more faithfully reflect local anatomy. For pharyngeal applications, it is essential to determine whether the formulation achieves adequate mucosal coverage, deposits within or near tonsillar crypts, accumulates in local lymphoid compartments, and clears in a manner consistent with repeat administration [60]. These data are particularly important because local benefit depends not only on reaching the target tissue but on doing so without uncontrolled spreading, prolonged persistence, or rapid washout by saliva and mucosal turnover. A third stage should evaluate safety in a way that mirrors intended clinical use. Because these systems may contact mucosal and lymphoid tissues directly, preclinical safety assessment should include repeated-dose local tolerability, inflammatory profiling, and evaluation of degradation, persistence, or tissue remodeling rather than relying only on generic systemic toxicology assumptions [61]. This is especially relevant for cationic, strongly mucoadhesive, or inorganic formulations, whose local exposure patterns may create risks not evident in short-term screening studies [62]. Only after a candidate has demonstrated acceptable formulation control, local distribution, and repeat-dose tolerability should early clinical translation be considered. Initial human studies are best designed as phase 0 or phase 1 investigations centered on measurable local endpoints, such as pharmacodynamic biomarker modulation, mucosal immune activation, imaging-defined deposition, and early viral-load effects, rather than broad efficacy claims [63]. Such a staged approach improves the chances that promising nanoparticle concepts will be tested in a manner consistent with real pharyngeal use and reduces the likelihood that attractive but poorly controlled formulations advance on the basis of incomplete evidence alone.

**Figure 5:**
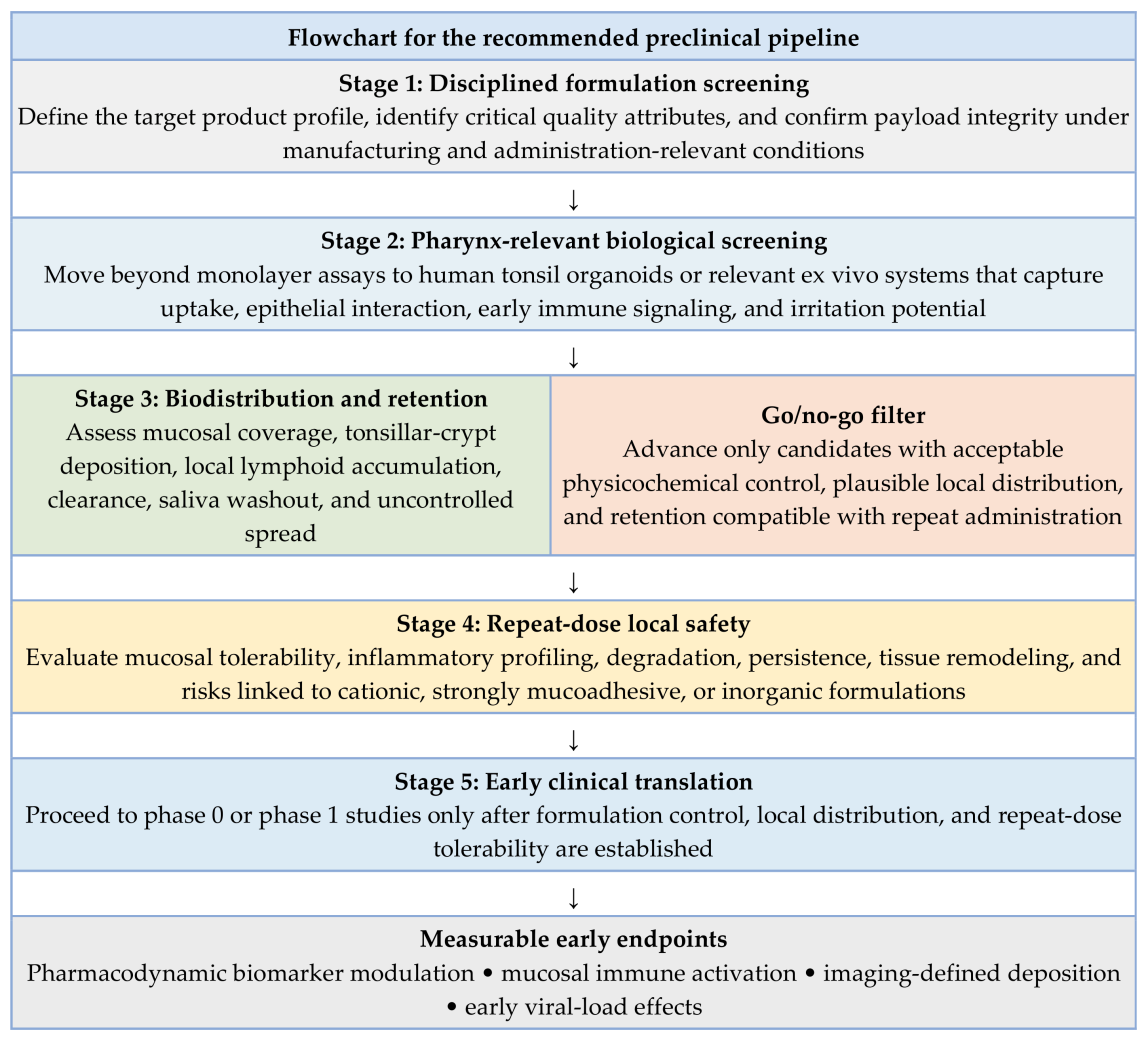
Flowchart summarizing the recommended preclinical pipeline for nanoparticle interventions in pharyngeal HPV. Candidate development should begin with disciplined formulation screening, including definition of a target product profile, identification of critical quality attributes, and confirmation of payload integrity under manufacturing and administration-relevant conditions. Promising formulations should then advance through pharynx-relevant biological screening, biodistribution and retention testing, and repeat-dose local safety assessment before phase 0 or phase 1 clinical translation. Early human studies should prioritize measurable local endpoints such as pharmacodynamic biomarker modulation, mucosal immune activation, imaging-defined deposition, and early viral-load effects rather than broad efficacy claims.

Representative preclinical efficacy and theranostic readouts that could support candidate advancement are illustrated conceptually in Figure 5.

## 5. Knowledge Gaps and Research Priorities

### 5.1. Oropharyngeal Microbiome and Nanoparticle Performance

Several additional research directions could substantially strengthen the field if they are developed with the same rigor applied to core formulation questions. One is the role of the oropharyngeal microbiome in nanoparticle performance [64]. The pharynx is not a passive delivery surface: microbial communities influence mucus properties, epithelial signaling, immune tone, and inflammatory balance, all of which may alter nanoparticle retention, degradation, uptake, and tolerability [65]. Repeated local dosing could also reshape the microbial environment in ways that affect both safety and antiviral efficacy. For this reason, future studies should consider whether specific materials or excipients disturb local microbial ecology and whether microbiome-aware formulation design could improve persistence or reduce irritation. A second opportunity lies in patient-centered dosage form design. Many preclinical systems are optimized for laboratory delivery rather than real-world use, yet success in the oropharynx will depend on practical issues such as taste, mouthfeel, ease of administration, dosing precision, and adherence to repeated use [66]. Comparative evaluation of sprays, rinses, films, and gels should therefore include not only physicochemical and pharmacologic metrics but also user acceptability and suitability for outpatient self-administration. This is especially relevant if local preventive or suppressive therapies are intended for extended use rather than one-time intervention [67]. A third promising direction is biomarker-guided development. Because pharyngeal HPV nanomedicines are unlikely to demonstrate broad clinical benefit early in development, progress will depend on sensitive local biomarkers that can confirm biological activity before large efficacy trials are feasible. Relevant endpoints may include HPV DNA or E6/E7 transcript burden, local cytokine patterns, mucosal antibody responses, tissue-resident memory markers, imaging-defined deposition, and short-term indicators of epithelial or lymphoid response. Incorporating biomarker strategy into formulation development would improve decision-making and enable more informative early-phase studies [68]. Combination therapy is another area with substantial potential. Rather than viewing vaccines, siRNA, CRISPR systems, and theranostics as isolated categories, future work could examine rational combinations such as local oncogene silencing paired with immune adjuvants, focal ablative therapy combined with antigen-releasing nanoparticle platforms, or local nanotherapy designed to complement systemic immunomodulation. These approaches may be especially useful when the goal is not merely delivery but also coordinated control of viral persistence, lesion biology, and local immune activation [69]. Computational and data-driven tools also deserve more attention. In silico modeling of mucus transport, particle deposition, and crypt penetration, as well as machine learning approaches for formulation optimization, could help reduce empirical trial and error and identify the parameter combinations most likely to succeed in pharyngeal tissue [70]. Site-specific targeting strategies for tonsillar crypt biology represent a further opportunity. The crypt microenvironment is both a barrier to and potential advantage for local delivery, and future materials could be designed with ligands, geometries, or rheological properties that improve deposition near crypt-associated epithelia or local immune structures [71]. Finally, translational research should consider implementation from the outset. Simpler, stable, and scalable products may ultimately have greater public-health impact than highly sophisticated systems that depend on complex manufacturing or cold-chain logistics. For that reason, cost, deployability, and regulatory classification should be treated not as late-stage concerns but as design variables that shape which nanoparticle concepts are worth advancing.

### 5.2. Comparative Structure–Property–Function Studies

The field now requires fewer proof-of-concept demonstrations and more disciplined comparative research. Despite increasing interest in nanomaterials for HPV-associated disease, direct evidence for pharyngeal HPV remains sparse, fragmented across platform classes, and often difficult to compare because model systems, analytical methods, and biological endpoints vary widely. As a result, the literature contains many promising claims but relatively few data sets that can guide rational selection of one material class over another. The most important research priority is therefore not to simply design new nanoparticle platforms but to define which physicochemical attributes consistently predict useful performance in the pharyngeal environment [72]. At present, this relationship remains underdeveloped. Particle size, surface charge, hydration behavior, stiffness, degradability, and ligand density are all discussed as relevant variables, yet studies rarely test them systematically under conditions that reflect saliva exposure, lymphoepithelial architecture, and repeated local dosing. Comparative studies using common assay conditions would do more to advance the field than another generation of highly customized formulations evaluated only in isolated in vitro settings [73].

### 5.3. Pharynx-Relevant Biological Models

A second major gap concerns biological models. Much of the available literature on nanomedicine still depends on conventional epithelial monolayers, simplified mucus surrogates, or non-pharyngeal tissues. These models are useful for initial screening, but they do not reproduce the anatomical and immunological features that make Waldeyer’s ring distinctive. Tonsillar crypts create confined geometries; the overlying epithelium is specialized and discontinuous; saliva is continuously present; and immune-cell interactions occur in close proximity to epithelial barriers. These features are central to vaccine delivery, local gene silencing, and any effort to modulate persistent HPV infection [74]. The development and wider adoption of human tonsil organoids, ex vivo tonsillar tissue systems, and more informative head-and-neck organoid models should therefore be treated as a priority rather than a specialized luxury. Such models are especially valuable because they allow material scientists to study deposition, penetration, immune activation, and toxicity in a human lymphoepithelial context that is far more relevant than generic cell culture systems. They may also help distinguish which apparent advantages of mucoadhesive or mucus-penetrating formulations are genuine and which are artifacts of oversimplified test conditions. Evidence from human tonsil organoid systems already shows their utility for modeling vaccine-driven immune responses and local cellular activation, underscoring their importance for platform evaluation [75].

### 5.4. Mucosal Immunology Endpoints

A third gap lies in the limited integration of mucosal immunology with materials design. Many vaccine-oriented nanoparticle studies still report serum antibody levels or generalized cellular responses without determining whether the formulation generates the local immune features most relevant to pharyngeal protection. For this anatomical site, durable benefit is likely to depend on secretory antibody production, antigen presentation within local lymphoid tissue, and the establishment of tissue-resident memory populations rather than on systemic responses alone. Recent reviews of mucosal vaccinology emphasize that correlates of protection at mucosal surfaces remain incompletely defined and that route, adjuvant, and delivery vehicle strongly shape local immunity [76]. Future pharyngeal HPV studies should therefore include more informative immunologic endpoints, such as local IgA-related readouts, dendritic-cell activation patterns, cytokine balance, and markers of tissue-resident memory rather than relying exclusively on standard peripheral measurements. This is particularly important because a formulation that appears to be immunogenic after intramuscular administration may not translate into meaningful protection when reformulated for local oropharyngeal delivery [77].

### 5.5. Standardized Materials Characterization

Materials characterization is another area in need of higher standards. In a field centered on structure–property–function logic, it remains surprisingly common for studies to underreport polydispersity, inter-batch variability, storage stability, release conditions, or the impact of saliva-like media on colloidal behavior. This weakens interpretation because biological outcomes cannot be linked confidently to material design. Future work should define a core set of critical quality attributes for each platform class, supported by orthogonal methods wherever possible [78]. For lipid nanoparticle platforms, this includes particle size distribution, encapsulation efficiency, ionization behavior, cargo integrity, and stability during storage and administration. For polymeric systems, molecular-weight distribution, degradation profile, mucoadhesive behavior, and residual-solvent control are equally important. Hybrid and theranostic systems require an even broader analytical package because interfacial architecture, coating stability, and triggerable functions can change independently during scale-up or storage. Without standardized reporting, claims of superiority across platforms will remain difficult to evaluate [79].

### 5.6. Dosage Form Design and Patient Acceptability

The dosage form itself is also an underexamined determinant of success. Nanoparticle platforms intended for the oropharynx will rarely be administered as idealized laboratory dispersions; they will likely need to be incorporated into sprays, rinses, films, gels, or other local systems that impose constraints on viscosity, spray pattern, patient comfort, sterility, and packaging compatibility. Yet, many publications treat the dosage form as a secondary issue and focus only on the nanoparticle core [80]. A material that performs well in a suspension may fail once incorporated into a clinically realistic spray or film because aggregation, release, and surface interactions change. Future studies should therefore compare clinically relevant dosage forms early in development, including evaluation of rheology, aerosolization or spreading, local retention, mucosal coverage, and user acceptability. Head-to-head comparisons of mucoadhesive versus mucus-penetrating strategies would be especially valuable because both are frequently proposed but rarely tested under common conditions that would reveal when one approach is preferable to the other.

### 5.7. Repeat-Dose Mucosal Safety and Lymphoid Exposure

Safety remains another central knowledge gap, particularly for repeated mucosal dosing and for platforms intended to contact lymphoid tissue directly. Much of the broader nanomedicine literature relies on systemic toxicology logic, but the pharyngeal setting raises distinct questions as to whether particles cause chronic irritation, alter local microbial balance, accumulate in crypt-associated tissue, or sustain inflammatory activation after repeated administration. These risks are especially relevant for cationic materials, persistent inorganic particles, and immunostimulatory payloads. The priority here is not merely to perform more toxicity studies but to design safety packages that reflect how the product will actually be used. Evaluations of repeat-dose local tolerability, persistence or clearance, cytokine profiling, and tissue remodeling markers should become standard in preclinical programs. More broadly, the literature on HPV nanotherapy increasingly recognizes that poor model selection and incomplete safety assessment are major reasons why the development of promising concepts stalls before clinical use [81].

### 5.8. Manufacturing, Regulatory Planning, and Early Clinical Endpoints

Finally, research priorities must extend beyond benchtop optimization to manufacturing and early clinical planning. A recurrent weakness in the literature is the assumption that translational questions can be deferred until efficacy is confirmed. In practice, manufacturability, comparability, sterilization strategy, raw-material control, and regulatory classification strongly influence which platform can progress. This issue is particularly important for hybrid systems, theranostics, and genome-editing formulations, whose complexity may outpace their translational justification. By contrast, the most valuable next-generation studies will likely be those that begin with a clear target product profile, define go/no-go criteria, and evaluate whether a formulation can be produced reproducibly at useful scale. From a clinical perspective, early trials should focus on measurable local endpoints—such as deposition, biomarker modulation, viral-load change, or mucosal immune activation—rather than broad efficacy claims. Overall, the field would benefit from a shift in culture from celebrating novelty alone to rewarding rigor, comparability, and translational discipline. If that shift occurs, nanoparticle research for pharyngeal HPV could move from a promising conceptual space to a genuinely actionable therapeutic and preventive pipeline [82,83].

### 5.9. Critical Review of the References Identified

In this section, we critically appraise the references used to support this review, distinguishing between sources that provide direct evidence for pharyngeal HPV nanomedicine and those that offer a broader methodological, immunological, formulation, or translational context. The analysis emphasizes the evidentiary weight of each citation by considering study type, anatomical relevance, clinical maturity, and transferability to the oropharyngeal setting. Because direct pharyngeal HPV nanoparticle data remain limited, many references are valuable as conceptual or platform-level anchors rather than definitive proof. Table 2 therefore helps clarify which claims are strongly supported, which rely on extrapolation, and where further targeted evidence is still required.

## 6. Future Directions

Future work should focus less on adding functionality and more on establishing design rules that are reproducible across laboratories. The most informative studies will be those that compare platform classes using standardized assays for particle size distribution, surface charge, loading, release, mucus interaction, and storage stability, followed by relating those measurements to biological outcomes such as tonsillar deposition, epithelial uptake, and immune activation [84]. The field also needs better pharynx-relevant model systems, especially organoid and ex vivo platforms that capture epithelium–immune interactions, and more careful analysis of dosage forms suitable for real clinical use [85]. Comparative studies of mucoadhesive versus mucus-penetrating strategies, repeated local dosing, and container-closure or sterilization effects would be particularly valuable [86]. In parallel, translational planning should begin earlier: quality-by-design frameworks, manufacturing feasibility, and regulatory classification should be considered during formulation selection rather than after proof-of-concept efficacy is achieved [87].

## 7. Conclusions

Nanoparticle materials offer a promising but still developing framework for addressing persistent gaps in the prevention, detection, and treatment of pharyngeal HPV infection. At present, the strongest case is for relatively simple, analytically tractable systems—especially lipid and polymeric carriers—designed either for mucosal vaccination or for localized delivery of nucleic acid therapeutics. These platforms align most closely with the requirements of reproducible fabrication, measurable critical quality attributes, and plausible local administration. More complex theranostic and genome-editing systems remain scientifically important, but their path to clinical application is longer because product complexity, safety assessment, and the regulatory burden increase sharply. Progress in this field will depend less on producing increasingly elaborate nanostructures than on generating convincing evidence that specific material properties lead to reliable performance in pharyngeal tissue and can be maintained during manufacturing, storage, and clinical use.

## Figures and Tables

**Table 1:** Nanoparticle platform comparison.

Platform	Representative Materials	Critical Physicochemical Attributes	Best-Matched Payloads	Characterization Priorities	Manufacturing and Scale-Up Considerations	Most Plausible Use Case in Pharyngeal HPV	Relative Translational Readiness
Lipid nanoparticle platforms (LNPs) [16]	Ionizable lipids, PEG lipids, cholesterol, helper phospholipids	Particle size distribution, polydispersity, pKa-dependent ionization, encapsulation efficiency, colloidal stability	mRNA, siRNA, selected small molecules	Dynamic light scattering, zeta potential, cryo-TEM, encapsulation and release assays, serum and mucus stability	Microfluidic or controlled-mixing reproducibility, raw-material consistency, sterilization compatibility, cold-chain or storage requirements	Mucosal vaccination and localized nucleic acid delivery	Relatively high for nucleic acid applications
Polymeric nanoparticle platforms [17]	PLGA, chitosan, hyaluronic acid, PEGylated polymers, thiolated polymers	Molecular weight distribution, surface chemistry, swelling behavior, mucoadhesion, degradation rate	Proteins, peptides, nucleic acids, small molecules	Size and morphology analysis, surface functional-group quantification, release kinetics, rheology or mucoadhesion testing, degradation profiling	Solvent selection and removal, batch-to-batch reproducibility, residual-solvent control, lyophilization or spray-drying feasibility	Mucoadhesive local delivery, protein or peptide vaccination, sustained tonsillar exposure	Moderate, with strong rationale for local delivery
Inorganic nanoparticle platforms [18]	Gold, iron oxide, silica	Core size and shape, crystallinity, surface coating density, optical or magnetic response, dissolution behavior	Imaging agents, photothermal or photodynamic payloads	Electron microscopy, X-ray methods, spectroscopy, photothermal conversion or relaxivity testing, protein-corona assessment	Surface-functionalization reproducibility, impurity control, long-term storage stability, more demanding safety packaging for repeated exposure	Lesion mapping and focal ablation in selected settings	Early for pharyngeal use; more exploratory than near-future
Hybrid or biomimetic nanoparticle platforms [19]	Lipid–polymer hybrids, membrane-coated particles, multifunctional composites	Interfacial architecture, coating integrity, multicomponent stability, targeting-ligand density, triggerable release behavior	Combined antigen-adjuvant systems, nucleic acids, theranostic payloads	Orthogonal multimodal characterization, coating validation, release profiling, biological corona studies, stress testing	Complex process control, analytical burden, more challenging comparability and quality-by-design implementation	Advanced immunotherapy and integrated theranostic concepts	Early but potentially high-impact if simplification is feasible

**Table 2:** Critical review of the references identified.

Citation	Type/Focus	Strengths	Limitations	Interpretation
Lechner [1]	Authoritative review of HPV-OPSCC	Comprehensive, mechanistic, clinically integrated	Narrative, non-systematic	Foundational framework; requires triangulation with primary data
Windon [2]	Epidemiologic trend analysis	Large cohort; clear demographic signal	Retrospective; assay variability	Strong descriptive evidence; causal drivers remain uncertain
Weerarathna [3]	Vaccine development review	Broad platform coverage	Preclinical focus	Useful landscape; clinical maturity limited
Orzell [4]	Clinical infectious disease overview	Solid clinical context	Not HPV-specific	Background only
Fossum [5]	Oropharyngeal anatomy/immunology	High anatomical relevance	Descriptive	Supports mechanistic sections
Zhou [6]	Mucosal immunity review	Mechanistic depth	Broad, non-HPV-specific	Strong conceptual anchor
Dobrovolskaia [7]	NP–immune interactions	Foundational immunotoxicology	Older platforms	Essential safety context
Pašti [8]	Analytical perspective	Method innovation	Indirect relevance	Peripheral methodological insight
Day [9]	HPV-OPSCC screening review	Balanced clinical framing	No trial data	Clarifies why screening remains unestablished
Gallus [10]	p16 diagnostic accuracy	Multicenter, clinically actionable	Imperfect surrogate	Supports diagnostic pathway discussion
Anaya-Saavedra [11]	HPV oral lesion challenges	Real-world clinical gaps	Narrative	Highlights diagnostic uncertainty
Lopes-Nunes [12]	Nanotherapy for HPV cancers	HPV-focused synthesis	Preclinical	Strong for opportunities/challenges
Sukhera [13]	Narrative review methodology	Clarifies rigor	Not disease-specific	Supports methodological transparency
Jiang [14]	AI histopathology	Advanced computational pathology	Different disease	Methodological relevance only
Jung [15]	LNPs for RNA therapeutics	Detailed platform science	Not HPV-specific	Strong for delivery platform section
Xu [16]	LNP composition/formulation	High formulation detail	General	Supports nanocarrier design discussion
Ensign [17]	Mucus barrier and NP transport	Classic mechanistic work	Dated	Still relevant for mucosal delivery barriers
Anselmo [18]	Inorganic NP translation	Regulatory insight	Inorganic focus	Useful for translational constraints
Firouzpour [19]	NP cancer delivery	Mechanistic overview	Broad	Platform-level relevance
Triantafyllopoulou [20]	Lipid/copolymer hybrids	Strong design-performance link	Sparse clinical data	Supports hybrid system rationale
Van de Casteele [21]	Pig mucosal LNP-saRNA	Large-animal relevance	Non-HPV pathogen	Strong translational evidence for mucosal LNPs
Subramanian [22]	Mucoadhesive nutraceuticals	Clear mucoadhesion principles	Nutraceutical focus	Mechanistic relevance only
Boyaka [23]	Mucosal vaccine overview	Foundational	Dated	Historical anchor; must be updated
Talks [24]	Waldeyer’s ring immunity	Direct anatomical relevance	Descriptive	Essential for tonsillar immunobiology
Wang [25]	Biomolecular interfaces	Targeting mechanisms	General	Supports nano-targeting section
Sarkar [26]	Adjuvant selection	Practical guidance	Limited HPV data	Useful for vaccine design
Chatterjee [27]	Mucoadhesive polymers	Mechanistic clarity	Preclinical	Supports mucosal formulation section
Kirkby [28]	CD4+ TRM cells	Mechanistic depth	Limited HPV data	Strong for TRM-centric vaccine rationale
Aldosari [29]	LNPs for RNA vaccines	COVID-era insights	Systemic focus	Platform relevance
Lange [30]	Human mucosal TRM	High immunologic relevance	Extrapolative	Supports durable mucosal immunity argument
Li [31]	HPV integration	Deep mechanistic insight	Cervical focus	Mechanistic parallels to OPSCC
Khari [32]	siRNA/mRNA HPV therapy	Nucleic acid therapeutics	Cervical-disease-specific	Conceptual relevance
Hariharan [33]	Local HNSCC delivery	Direct site relevance	Heterogeneous modalities	Supports localized therapy section
Zhang [34]	Lipid carriers for mRNA	Detailed carrier science	General	Strong for delivery platform discussion
Vila [35]	Saliva immunity	Diagnostic/immune relevance	Non-HPV	Peripheral but useful
Chakraborty [36]	CRISPR therapeutics	Up-to-date clinical landscape	Broad	Supports genome-editing section
Lummerstorfer [37]	Non-viral CRISPR carriers	Delivery innovation	Preclinical	Platform relevance
Ortiz-Bueno [38]	Genome editing ethics	Strong translational ethics	Non-specific	Supports ethical considerations
Wu [39]	Biopolymer sustained delivery	Innovative local delivery	Preclinical	Supports controlled-release section
Firmansyah [40]	Film-forming gels	Systematic formulation evidence	Skin-focused	Limited mucosal transferability
Maraie [41]	Buccal mucoadhesive film	Practical design	Non-oncologic	Supports mucoadhesion principles
Cruz [42]	Viscosity mitigation	Predictive formulation	Biotherapeutic focus	Indirect relevance
Zoeller [43]	Hydrogels in biomedicine	Clinical trial relevance	Broad	Supports hydrogel-based delivery
Kelkar [44]	Theranostics	Foundational	Dated	Historical context
Vines [45]	Gold NP photothermal	Mechanistic clarity	Non-HPV	Supports alternative modalities
López [46]	HPV detection review	Technology-focused	Narrative	Strong diagnostic landscape mapping
Dzuvor [47]	Protein NP antibacterial	Mechanistic innovation	Non-oncologic	Platform relevance
Patel [48]	HPV status & salvage surgery	Clinically meaningful	Retrospective	Supports prognostic discussion
Zhang [49]	Mucosal immunity & vaccines	Up-to-date	General	Strong conceptual relevance
Okusanya [50]	Oncology dose optimization	Methodologically strong	Non-specific	Supports trial design section
Zhang [51]	Respiratory mucosal immunity	Mechanistic depth	Respiratory focus	Conceptual parallels
Yadav [52]	CRISPR theranostics	Integrative	Preclinical	Supports emerging modalities
Massoni-Badosa [53]	Tonsil cell atlas	High-resolution human data	Descriptive	Essential for tonsillar microenvironment
Goldwater [54]	Systemic vs. mucosal immunity	Immunologic framing	Controversial	Peripheral cautionary context
Wen [55]	Reproducibility critique	Critical meta-perspective	Non-specific	Essential for evidence-quality framing
Bas [56]	Biosimilar formulation	Regulatory insight	Indirect	Supports formulation constraints
Wagar [57]	Tonsil organoids	Human-relevant model	Not HPV-specific	Strong experimental platform
Huang [58]	Tonsil innate immunity	Direct tissue data	Pediatric; non-HPV	Supports innate immunity section
Shah [59]	Preclinical formulation	Practical	General	Background formulation guidance
Kim [60]	Tonsil epithelial organoids	Viral infection model	SARS-CoV-2	Platform relevance
Onodera [61]	Inhaled particles & immunity	Safety relevance	Respiratory	Supports mucosal toxicology
Green [62]	Vaccine toxicology	Comprehensive	General	Supports safety section
Catani [63]	Biomarker enrichment	Trial optimization	Non-specific	Supports precision trial design
Di Spirito [64]	Micro/nanoplastics oral cavity	Exposure & health	Environmental	Supports mucosal NP safety
Mafe [65]	Microbiome & anticancer therapy	Mechanistic	General	Supports microbiome–therapy interactions
Limenh [66]	Dosage form preference	Patient-centered	Non-oncology	Supports acceptability considerations
Antoniou [67]	Biomarker-guided trials	Practical	General	Supports trial design
Shin [68]	Virotherapy immune evasion	Mechanistic	Non-HPV	Supports immunotherapy section
Hornick [69]	AI formulation design	Cutting-edge	Disease-agnostic	Supports AI-enabled formulation
Sethi [70]	HPV tonsillar carcinoma biology	Site-specific	Heterogeneous data	Strong mechanistic anchor
Aarons [71]	Scaling-out interventions	Implementation science	General	Supports translational scalability
Xu [72]	Nanomedicine for HPV-HNC	HPV-specific	Preclinical	Strong translational roadmap
Davies Forsman [73]	Salivary TDM	Methodological	Antimicrobial	Peripheral relevance
Bogoslowski [74]	Lymphoid organoids	Platform	Non-HPV	Supports experimental modeling
Hill [75]	Airway mucus physiology	Mechanistic	Respiratory	Supports mucosal barrier discussion
Salam [76]	Next-gen vaccines	Modern immunology	General	Strong conceptual relevance
Rojo-Fernandezv [77]	Human mucosal/systemic immunity	Human data	SARS-CoV-2	Supports mucosal vaccine rationale
Hasan [78]	NP delivery for oral cancer	Mechanistic	Non-HPV	Supports delivery barrier discussion
Abdelhakim [79]	Oral dosage design	Translational	General	Supports formulation section
Graham [80]	Occupational exposure limits	Safety	Occupational	Peripheral
Desai [81]	NP therapeutics & regulation	Clinical/regulatory	General	Supports regulatory landscape
Sharma [82]	Nanotherapeutics for HPV cancers	HPV-focused	Preclinical	Strong balanced synthesis
Wang [83]	Low-liver LNP HPV vaccine	Mechanistic innovation	Animal	Promising preclinical evidence
Yazdani [84]	AI-designed smart nanocarriers	Cutting-edge	Preclinical	Supports future directions
Porfiryeva [85]	NP–mucin hydrogel interactions	Direct mucus data	In vitro	Supports mucosal delivery constraints
Adepu [86]	Controlled drug delivery	Broad	General	Background
Yang [87]	QbD in pharma	Industrial rigor	General	Supports manufacturing/quality section

## Data Availability

No new data were created or analyzed in this study. Data sharing is not applicable to this article.

## Abbreviations

The following abbreviations are used in this manuscript: ASO, antisense oligonucleotide; CRISPR, clustered regularly interspaced short palindromic repeats; GLP, good laboratory practice; GMP, good manufacturing practice; HPV, human papillomavirus; LNP, lipid nanoparticle; NP, nanoparticle; OPSCC, oropharyngeal squamous cell carcinoma; PEG, poly(ethylene glycol); PLGA, poly(lactic-co-glycolic acid); QbD, quality by design; RNP, ribonucleoprotein; TRM, tissue-resident memory.

## References

[1] Lechner M., Liu J., Masterson L., Fenton T.R. (2022). HPV-associated oropharyngeal cancer: Epidemiology, molecular biology and clinical management. Nat. Rev. Clin. Oncol..

[2] Windon M.J., D’Souza G., Rettig E.M., Westra W.H., van Zante A., Wang S.J., Ryan W.R., Mydlarz W.K., Ha P.K., Miles B.A. (2018). Increasing prevalence of human papillomavirus-positive oropharyngeal cancers among older adults. Cancer.

[3] Weerarathna I.N., Doelakeh E.S., Kiwanuka L., Kumar P., Arora S. (2024). Prophylactic and therapeutic vaccine development: Advancements and challenges. Mol. Biomed..

[4] Orzell S., Suryadevara A. (2018). Pharyngitis and Pharyngeal Space Infections: Fever, sore throat, difficulty swallowing. Introduction to Clinical Infectious Diseases.

[5] Fossum C.C., Chintakuntlawar A.V., Price D.L., Garcia J.J. (2017). Characterization of the oropharynx: Anatomy, histology, immunology, squamous cell carcinoma and surgical resection. Histopathology.

[6] Zhou X., Wu Y., Zhu Z., Lu C., Zhang C., Zeng L., Xie F., Zhang L., Zhou F. (2025). Mucosal immune response in biology, disease prevention and treatment. Signal Transduct. Target. Ther..

[7] Dobrovolskaia M.A., Shurin M., Shvedova A.A. (2016). Current understanding of interactions between nanoparticles and the immune system. Toxicol. Appl. Pharmacol..

[8] Pašti I.A., Mentus S.V. (2021). “Spot the difference”—Just a game or the game-changer?. Joule.

[9] Day A.T., Fakhry C., Tiro J.A., Dahlstrom K.R., Sturgis E.M. (2020). Considerations in Human Papillomavirus–Associated Oropharyngeal Cancer Screening: A Review. JAMA Otolaryngol. Head Neck Surg..

[10] Gallus R., Nauta I.H., Marklund L., Rizzo D., Crescio C., Mureddu L., Tropiano P., Delogu G., Bussu F. (2023). Accuracy of p16 IHC in Classifying HPV-Driven OPSCC in Different Populations. Cancers.

[11] Anaya-Saavedra G., Castillejos-García I., Vázquez-Garduño M. (2026). Diagnostic challenges and clinical management gaps in HPV-related oral lesions. Front. Oral Health.

[12] Lopes-Nunes J., Oliveira P.A., Cruz C. (2024). Nanotherapy for human papillomavirus-associated cancers: Breakthroughs and challenges. Trends Pharmacol. Sci..

[13] Sukhera J. (2022). Narrative Reviews: Flexible, Rigorous, and Practical. J. Grad. Med. Educ..

[14] Jiang X., Li P., Chen Z., Yao H., Jia H., Wang T., Tang X. (2026). Deep learning-based quantitative histopathology of endoscopic biopsies in Crohn’s disease: A retrospective cross-sectional validation study. Front. Immunol..

[15] Jung H.N., Lee S.Y., Lee S., Youn H., Im H.J. (2022). Lipid nanoparticles for delivery of RNA therapeutics: Current status and the role of in vivo imaging. Theranostics.

[16] Xu S., Hu Z., Song F., Xu Y., Han X. (2025). Lipid nanoparticles: Composition, formulation, and application. Mol. Ther. Methods Clin. Dev..

[17] Ensign L.M., Cone R., Hanes J. (2012). Oral drug delivery with polymeric nanoparticles: The gastrointestinal mucus barriers. Adv. Drug Deliv. Rev..

[18] Anselmo A.C., Mitragotri S. (2015). A Review of Clinical Translation of Inorganic Nanoparticles. AAPS J..

[19] Firouzpour H., Najafi F., Mohammadgholi A., Alizadegan D., Beram F.M., Akhtari N., Zoghi M., Ansar N., Yaraki M.T. (2026). Nanoparticle-based drug delivery systems for effective cancer treatment: Mechanisms and applications. Next Nanotechnol..

[20] Triantafyllopoulou E., Selianitis D., Chountoulesi M., Karayianni M., Valsami G., Pispas S., Pippa N. (2026). Lipid/copolymer hybrid drug delivery systems: From design to performance. Adv. Colloid Interface Sci..

[21] Van de Casteele I., Plovyt M., Stuchlíková M., Lanssens M., Verschueren B., Denon Q., Van der Meeren P., McCafferty S., Gitsels A., Cornillie P. (2026). Mucosal administration of lipid nanoparticles containing self-amplifying mRNA induces local uptake and expression in a pig model as a potential vaccination platform against STIs. Drug Deliv. Transl. Res..

[22] Subramanian P. (2021). Mucoadhesive Delivery System: A Smart Way to Improve Bioavailability of Nutraceuticals. Foods.

[23] Boyaka P.N., McGhee J.R., Czerkinsky C., Mestecky J. (2005). Mucosal Vaccines: An Overview. Mucosal Immunology.

[24] Talks B.J., Mather M.W., Chahal M., Coates M., Clatworthy M.R., Haniffa M. (2024). Mapping Human Immunity and the Education of Waldeyer’s Ring. Annu. Rev. Genom. Hum. Genet..

[25] Wang Z., Dai L., Zhu Z., Shang X. (2026). Biomolecular Interfaces in Targeted Nano-Drug Delivery: Molecular Recognition, Signaling Modulation, and Translational Pathways. Biomolecules.

[26] Sarkar I., Garg R., van Drunen Littel-van den Hurk S. (2019). Selection of adjuvants for vaccines targeting specific pathogens. Expert Rev. Vaccines.

[27] Chatterjee B., Amalina N., Sengupta P., Mandal U.K. (2017). Mucoadhesive polymers and their mode of action: A recent update. J. Appl. Pharm. Sci..

[28] Kirkby M., Veldhoen M. (2025). CD4+ tissue-resident memory T cells and their role in immunity. Immunol. Cell Biol..

[29] Aldosari B.N., Alfagih I.M., Almurshedi A.S. (2021). Lipid Nanoparticles as Delivery Systems for RNA-Based Vaccines. Pharmaceutics.

[30] Lange J., Rivera-Ballesteros O., Buggert M. (2022). Human mucosal tissue-resident memory T cells in health and disease. Mucosal Immunol..

[31] Li J., Li S. (2025). From Viral Infection to Genome Reshaping: The Triggering Role of HPV Integration in Cervical Cancer. Int. J. Mol. Sci..

[32] Khari M., Jain N., Kaul S., Pandey M., Sharma N. (2025). siRNA and mRNA-Based Preventive and Therapeutic Strategies for HPV-Induced Cervical Cancer. Adv. Pharm. Bull..

[33] Hariharan A., Tran S.D. (2023). Localized Drug Delivery Systems: An Update on Treatment Options for Head and Neck Squamous Cell Carcinomas. Pharmaceutics.

[34] Zhang W., Jiang Y., He Y., Boucetta H., Wu J., Chen Z., He W. (2023). Lipid carriers for mRNA delivery. Acta Pharm. Sin. B.

[35] Vila T., Rizk A.M., Sultan A.S., Jabra-Rizk M.A. (2019). The power of saliva: Antimicrobial and beyond. PLoS Pathog..

[36] Chakraborty C., Bhattacharya M., Das A., Agoramoorthy G., Lee S.S. (2026). CRISPR-Cas9-mediated therapeutics: Current clinical trials and therapy approval landscape to treat human diseases. Mol. Ther. Nucleic Acids.

[37] Lummerstorfer M., Lächelt U. (2026). Non-Viral CRISPR carriers: Transient delivery with lasting effects. Drug Deliv..

[38] Ortiz-Bueno M., Zinghirino F., Serra P.P., Paschoudi K., Montoliu L., Atilla E., Luo Y., Cavazza A., Lederer C.W., Benabdellah K. (2026). From Bench to Bedside: Ethical and Clinical Best Practices for Genome Editing Applications. Int. J. Mol. Sci..

[39] Wu J., Shaidani S., Theodossiou S.K., Hartzell E.J., Kaplan D.L. (2022). Localized, on-demand, sustained drug delivery from biopolymer-based materials. Expert Opin. Drug Deliv..

[40] Firmansyah F., Budiman A., Muchtaridi M., Chabib L., Syaputri F.N., Afandi F., Elamin K.M., Mohammed A.F.A., Wathoni N. (2026). Film-Forming Gels for Topical Drug Delivery: A Systematic Review of the Effects of Formulation on Film Performance, Drug Release, and Skin Permeation. Drug Des. Dev. Ther..

[41] Maraie N.K., Wannas A.N. (2026). Design, optimization and characterization of mucoadhesive buccal film as a novel delivery system for doxazosin mesylate. Saudi Pharm. J..

[42] Cruz M.A., Blanco M., Ekladious I. (2025). Mechanistic and predictive formulation development for viscosity mitigation of high-concentration biotherapeutics. mAbs.

[43] Zoeller K., To D., Bernkop-Schnuerch A. (2025). Biomedical applications of functional hydrogels: Innovative developments, relevant clinical trials and advanced products. Biomaterials.

[44] Kelkar S.S., Reineke T.M. (2011). Theranostics: Combining imaging and therapy. Bioconjug. Chem..

[45] Vines J.B., Yoon J.H., Ryu N.E., Lim D.J., Park H. (2019). Gold Nanoparticles for Photothermal Cancer Therapy. Front. Chem..

[46] López F., Bree R.d., Sreeram M.P., Nuyts S., Rodrigo J.P., Rao K.N., Saba N.F., Bradford C., Forastiere A., Kowalski L.P. (2026). HPV Detection in Oropharyngeal Cancer: A Narrative Review of Diagnostic and Emerging Molecular Approaches. Diagnostics.

[47] Dzuvor C.K.O., Shen H.H., Haritos V.S., He L. (2024). Coassembled multicomponent protein nanoparticles elicit enhanced antibacterial activity. ACS Nano.

[48] Patel T.R., Lee S., Tajudeen B.A., Stenson K., Bhayani M., Al-Khudari S. (2022). Association of HPV status with survival after surgical salvage of oropharyngeal cancers. Am. J. Otolaryngol..

[49] Zhang Z., Hong W., Zhang Y., Li X., Que H., Wei X. (2025). Mucosal immunity and vaccination strategies: Current insights and future perspectives. Mol. Biomed..

[50] Okusanya O.O., Patilea-Vrana G.I., Sireci A., Iasonos A., Davidson B.A., Liu J., Shord S.S., LoRusso P.M., Yap T.A. (2025). FDA-AACR Strategies for Optimizing Dosages for Oncology Drug Products: Early-Phase Trials Using Innovative Trial Designs and Biomarkers. Clin. Cancer Res..

[51] Zhang Y., Song Z., Hong W., He X., Wei X. (2026). Respiratory mucosal immunity: Biological functions, diseases, prevention and therapy. Genes Dis..

[52] Yadav N., Narang J., Chhillar A.K., Rana J.S. (2021). CRISPR: A new paradigm of theranostics. Nanomedicine.

[53] Massoni-Badosa R., Aguilar-Fernández S., Nieto J.C., Soler-Vila P., Elosua-Bayes M., Marchese D., Kulis M., Vilas-Zornoza A., Bühler M.M., Rashmi S. (2024). An atlas of cells in the human tonsil. Immunity.

[54] Goldwater P.N., Gorczynski R.M., Lindley R.A., Steele E.J. (2025). Sudden Infant Death Syndrome in the Context of the Future of Vaccination, and the Question of Systemic or Mucosal Immunity. Med. Res. Arch..

[55] Wen H., Wang H.Y., He X., Wu C.I. (2018). On the low reproducibility of cancer studies. Natl. Sci. Rev..

[56] Bas T.G. (2025). Innovative Formulation Strategies for Biosimilars: Trends Focused on Buffer-Free Systems, Safety, Regulatory Alignment, and Intellectual Property Challenges. Pharmaceuticals.

[57] Wagar L.E., Salahudeen A., Constantz C.M., Wendel B.S., Lyons M.M., Mallajosyula V., Jatt L.P., Adamska J.Z., Blum L.K., Gupta N. (2021). Modeling human adaptive immune responses with tonsil organoids. Nat. Med..

[58] Huang Q., Hua H., Li W., Chen X., Cheng L. (2020). Simple hypertrophic tonsils have more active innate immune and inflammatory responses than hypertrophic tonsils with recurrent inflammation in children. J. Otolaryngol. Head Neck Surg..

[59] Shah S.M., Jain A.S., Kaushik R., Nagarsenker M.S., Nerurkar M.J. (2014). Preclinical formulations: Insight, strategies, and practical considerations. AAPS PharmSciTech.

[60] Kim H.K., Kim H., Lee M.K., Choi W.H., Jang Y., Shin J.S., Park J.Y., Bae D.H., Hyun S.I., Kim K.H. (2022). Generation of human tonsil epithelial organoids as an ex vivo model for SARS-CoV-2 infection. Biomaterials.

[61] Onodera A. (2026). Biological Retention and Accumulation of Inhaled Environmental Particles Disrupt Immune Homeostasis: Implications for Chronic Lung Disease. Int. J. Environ. Med..

[62] Green M.D., Al-Humadi N.H. (2017). Preclinical Toxicology of Vaccines. A Comprehensive Guide to Toxicology in Nonclinical Drug Development.

[63] Catani G., Morchón-Araujo D., Mirallas O., Sánchez-Pérez V., Nuciforo P., Villacampa G., Dienstmann R., Vivancos A., Garralda E., Hernando-Calvo A. (2025). Optimizing early-phase immunotherapy trials: The role of biomarker enrichment strategies. Front. Immunol..

[64] Di Spirito F., Folliero V., Di Palo M.P., De Benedetto G., Aulisio L., Martina S., Rinaldi L., Franci G. (2025). Micro- and Nanoplastics and the Oral Cavity: Implications for Oral and Systemic Health, Dental Practice, and the Environment—A Narrative Review. J. Funct. Biomater..

[65] Mafe A.N., Büsselberg D. (2025). Microbiome Integrity Enhances the Efficacy and Safety of Anticancer Drug. Biomedicines.

[66] Limenh L.W., Tessema T.A., Simegn W., Ayenew W., Bayleyegn Z.W., Sendekie A.K., Chanie G.S., Fenta E.T., Beyna A.T., Kasahun A.E. (2024). Patients’ Preference for Pharmaceutical Dosage Forms: Does It Affect Medication Adherence? A Cross-Sectional Study in Community Pharmacies. Patient Prefer. Adherence.

[67] Antoniou M., Kolamunnage-Dona R., Wason J., Bathia R., Billingham C., Bliss J.M., Brown L.C., Gillman A., Paul J., Jorgensen A.L. (2019). Biomarker-guided trials: Challenges in practice. Contemp. Clin. Trials Commun..

[68] Shin D.H., Nguyen T., Ozpolat B., Lang F., Alonso M., Gomez-Manzano C., Fueyo J. (2021). Current strategies to circumvent the antiviral immunity to optimize cancer virotherapy. J. Immunother. Cancer.

[69] Hornick T., Mao C., Koynov A., Yawman P., Thool P., Salish K., Giles M., Nagapudi K., Zhang S. (2024). In silico formulation optimization and particle engineering of pharmaceutical products using a generative artificial intelligence structure synthesis method. Nat. Commun..

[70] Sethi S. (2025). Defining the Molecular Intricacies of Human Papillomavirus-Associated Tonsillar Carcinoma. Cancer Control.

[71] Aarons G.A., Sklar M., Mustanski B., Benbow N., Brown C.H. (2017). “Scaling-out” evidence-based interventions to new populations or new health care delivery systems. Implement. Sci..

[72] Xu Q., Chen Y., Jin Y., Wang Z., Dong H., Kaufmann A.M., Albers A.E., Qian X. (2022). Advanced Nanomedicine for High-Risk HPV-Driven Head and Neck Cancer. Viruses.

[73] Davies Forsman L., Kim H.Y., Nguyen T.A., Alffenaar J.C. (2024). Salivary Therapeutic Drug Monitoring of Antimicrobial Therapy: Feasible or Futile?. Clin. Pharmacokinet..

[74] Bogoslowski A., Ren J., Quintard C., Penninger J.M. (2025). Organoid Models of Lymphoid Tissues. Organoids.

[75] Hill D.B., Button B., Rubinstein M., Boucher R.C. (2022). Physiology and pathophysiology of human airway mucus. Physiol. Rev..

[76] Salam M.A., Al-Amin M.Y., Sudarshan K., Lynch A., Reyes V., Stevenson M. (2026). Next-Generation Vaccines Leveraging T Cell-Centric Design, Mucosal Immunity, and Trained Innate Immunity for Respiratory and Enteric Pathogens. Vaccines.

[77] Rojo-Fernandez A., Aslam S., Alam F., Escalera A., Villamandos V., Catón B., Megías G., Gonzalez A., Buzón-Martín L., Ayllon J. (2025). Systemic and mucosal immune signatures of protection against SARS-CoV-2 transmission in humans. Cell Rep. Med..

[78] Hasan N., Aftab M., Ullah M., Nguyen P.T., Agustina R., Djabir Y.Y., Tockary T.A., Uchida S. (2025). Nanoparticle-based drug delivery system for Oral Cancer: Mechanism, challenges, and therapeutic potential. Results Chem..

[79] Abdelhakim H.E., Awad A. (2025). From Material to Medicine: Translational Frontiers in Dosage Form Design for Oral Administration. Pharmaceutics.

[80] Graham J.C., Hillegass J., Schulze G. (2020). Considerations for setting occupational exposure limits for novel pharmaceutical modalities. Regul. Toxicol. Pharmacol..

[81] Desai N., Rana D., Patel M., Bajwa N., Prasad R., Vora L.K. (2025). Nanoparticle Therapeutics in Clinical Perspective: Classification, Marketed Products, and Regulatory Landscape. Small.

[82] Sharma U., Shekhar H., Sharma B., Sahu A., Haque S., Mathkor D.M., Kaur D., Tuli H.S., Mishra A., Ahmad F. (2025). Emerging nanotherapeutics for HPV-associated cancers: Current advances, challenges, and future directions. Discov. Oncol..

[83] Wang X., Tian Z., Wang M., Cai X., Yang S., Zeng J., Fang Y., Bai X., Wang P., Sun Y. (2025). Low-liver-accumulation lipid nanoparticles enhance the efficacy and safety of HPV therapeutic tumor vaccines. J. Transl. Med..

[84] Yazdani S., Mozaffarian M., Pazuki G., Hadidi N. (2026). Artificial intelligence-assisted design and optimization of stimuli-responsive nanocarriers for smart drug delivery. Mater. Today Bio.

[85] Porfiryeva N.N., Zlotver I., Sosnik A. (2025). Combined Effect of Size and Charge on the Interaction of Nanoparticles with Mucus-Mimicking Mucin Hydrogels. Pharmaceuticals.

[86] Adepu S., Ramakrishna S. (2021). Controlled Drug Delivery Systems: Current Status and Future Directions. Molecules.

[87] Yang S., Hu X., Zhu J., Zheng B., Bi W., Wang X., Wu J., Mi Z., Wu Y. (2025). Aspects and Implementation of Pharmaceutical Quality by Design from Conceptual Frameworks to Industrial Applications. Pharmaceutics.

